# External Guide Sequence Effectively Suppresses the Gene Expression and Replication of Herpes Simplex Virus 2

**DOI:** 10.3390/molecules29092052

**Published:** 2024-04-29

**Authors:** Bin Yan, Yujun Liu, Yuan-Chuan Chen, Fenyong Liu

**Affiliations:** 1 School of Public Health, University of California, Berkeley, CA 94720, USA; 2 Program in Comparative Biochemistry, University of California, Berkeley, CA 94720, USA

**Keywords:** RNase P, herpes simplex virus 2, antisense, gene targeting, antiviral, gene therapy

## Abstract

Ribonuclease P (RNase P) complexed with an external guide sequence (EGS) represents a promising nucleic acid-based gene targeting approach for gene expression knock-down and modulation. The RNase P-EGS strategy is unique as an EGS can be designed to basepair any mRNA sequence and recruit intracellular RNase P for hydrolysis of the target mRNA. In this study, we provide the first direct evidence that the RNase P-based approach effectively blocks the gene expression and replication of herpes simplex virus 2 (HSV-2), the causative agent of genital herpes. We constructed EGSs to target the mRNA encoding HSV-2 single-stranded DNA binding protein ICP8, which is essential for viral DNA genome replication and growth. In HSV-2 infected cells expressing a functional EGS, ICP8 levels were reduced by 85%, and viral growth decreased by 3000 folds. On the contrary, ICP8 expression and viral growth exhibited no substantial differences between cells expressing no EGS and those expressing a disabled EGS with mutations precluding RNase P recognition. The anti-ICP8 EGS is specific in targeting ICP8 because it only affects ICP8 expression but does not affect the expression of the other viral immediate-early and early genes examined. This study shows the effective and specific anti-HSV-2 activity of the RNase P-EGS approach and demonstrates the potential of EGS RNAs for anti-HSV-2 applications.

## 1. Introduction

Herpes simplex virus 2 (HSV-2) is the causative agent of genital herpes, a common sexually transmitted disease [1,2]. Moreover, this virus is one of the leading causes of potentially life-threatening neonatal encephalitis in newborns. Developing novel antiviral compounds and new strategies is critical for eliminating HSV-2 infection and its associated diseases.

**Figure 1:**
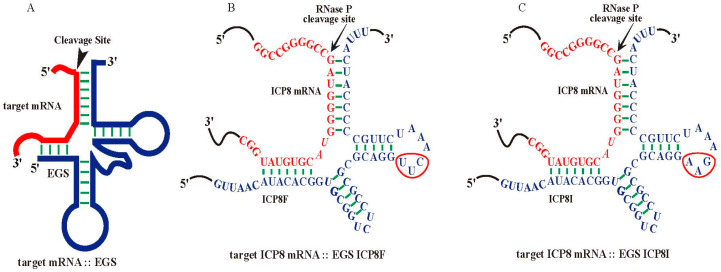
Substrate complexes of EGSs with a target mRNA for RNase P. (**A**) A complex of an EGS and a target mRNA resembling a pre-tRNA substrate. (**B**,**C**) Complexes of HSV-2 ICP8 mRNA (in red) and functional EGS ICP8F and inactive EGS ICP8I (in blue), respectively. The three mutated positions precluding RNase P recognition are circled. The arrowhead marks the RNase P cleavage site at the ICP8 sequence, which is 51 nucleotides downstream from the ICP8 translation initiation site. The open reading frame (ORF) UL29 contains the ICP8 coding sequence from the complementary strand between nucleotide positions 58,908 and 62,495 of the genome sequence of HSV-2 G strain (accession number OM370995.1).

Nucleic acid-based molecules represent promising therapeutic strategies by targeting specific mRNA sequences [3,4,5,6]. Ribonuclease P (RNase P) catalyzes a hydrolysis reaction to remove the leader sequence of a precursor tRNA (pre-tRNA) [7,8,9]. Enzyme-substrate interaction studies showed that RNase P mainly recognizes the overall structure instead of the nucleotide sequences of the pre-tRNA substrates (Figure 1A). Thus, short RNA molecules, termed external guide sequences (EGSs), can be designed to guide RNase P to cleave a target mRNA, such as a virus-encoded mRNA, provided that the EGS-mRNA complex looks like a pre-tRNA recognizable by RNase P [10,11] (Figure 1). In the EGS gene targeting strategy, a custom-designed EGS contains sequences resembling the T-stem, T-loop, and variable region of a tRNA (Figure 1). In addition, the EGS also contains “targeting” sequences complementary to the target mRNA sequence. The resulting complex between an EGS and a target mRNA resembles a pre-tRNA substrate, inducing RNase P to cleave the target mRNA [10,11]. EGSs have been shown as effective agents in inhibiting gene expression and replication of several human viruses, including HIV, herpes simplex virus 1 (HSV-1), human cytomegalovirus (HCMV), and hepatitis B virus (HBV) in cultured cells [12,13,14,15,16]. However, it has not been reported if EGSs also function for antiviral purposes against HSV-2.

In this study, we investigated the potential usage of EGSs as a gene-targeting agent for anti-HSV-2 applications. A functional EGS, ICP8F, was constructed to target the mRNA of HSV-2 ICP8, which encodes a single-stranded DNA binding protein essential for viral DNA genome replication [1,17]. Our study provides the first direct evidence of using EGSs for anti-HSV-2 therapy.

## 2. Results

### 2.1. In Vitro RNase P Cleavage of ICP8 mRNA Sequence Guided by EGS

Functional EGS ICP8F was derived from tRNA^SER^ [18,19] to target an ICP8 mRNA sequence with the RNase P cleavage site located 51 nucleotides downstream from the translation initiation site of ICP8 (Figure 1B). The open reading frame (ORF) UL29 contains the ICP8 coding sequence from the complementary strand between nucleotide positions 58,908 and 62,495 of the genome sequence of HSV-2 G strain (accession number OM370995.1). The target sequence appeared to be exposed because our experiments showed that this region was substantially modified by dimethyl sulfate (DMS) in HSV-2 infected cells. Thus, the target mRNA sequence is potentially accessible for EGS binding in viral-infected cells.

ICP8F contained a 5′ and 3′ sequence binding to the ICP8 mRNA sequence, flanked with a sequence resembling the variable region, T-loop, and T stem of tRNA^SER^ (Figure 1A). We also constructed a control inactive EGS, ICP8I, which was derived from ICP8F and contained three point mutations (from 5′-UUC-3′ to 5′-AAG-3′) in the conserved T-loop region known to disrupt RNase P recognition [13,18,19,20] (Figure 1C). We assayed the in vitro cleavage of a 38-nucleotide-long ICP8 mRNA substrate, sub-icp8, by RNase P (from HeLa cells) directed by ICP8F and ICP8I. ICP8F actively guided human RNase P in cleaving the substrate sub-icp8 (Table 1). As expected, ICP8I was not active in guiding RNase P cleavage, due to the mutations disrupting RNase P interaction. ICP8I contained an identical complementary sequence to sub-icp8 as ICP8F (Figure 1B,C) and also bound to sub-icp8 (K_d_ = 1.5 ± 0.3 µM) just as well as ICP8F (K_d_ = 1.5 ± 0.3 µM) (Table 1). Thus, ICP8I, which shares similar structures with ICP8F but with inactivated EGS activity, can serve as a control to exclude antisense or other unspecific effects of the EGS.

### 2.2. EGS Expression in Human Cells

**Figure 2:**
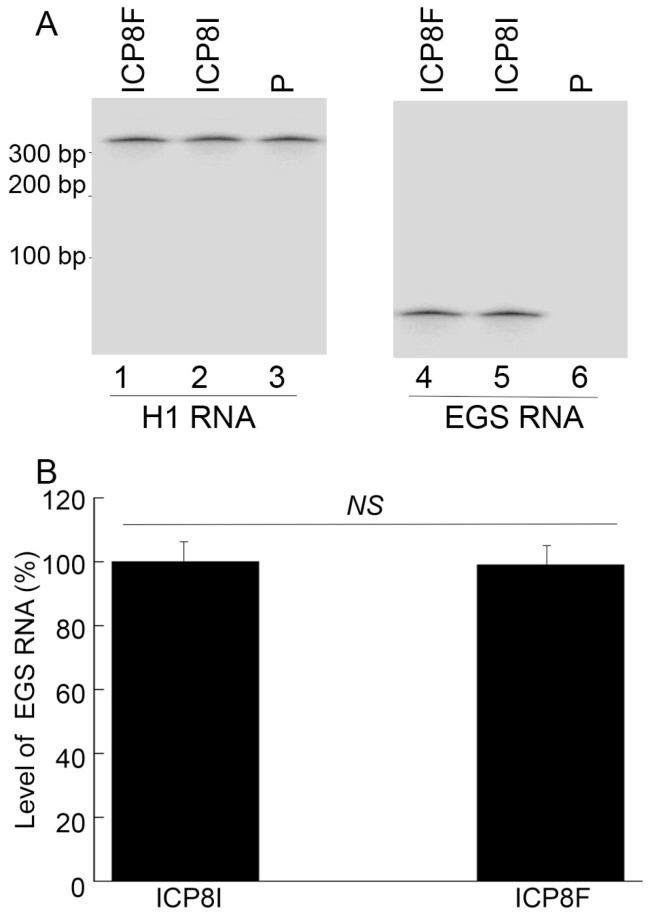
EGS RNA expression in cells detected by Northern blot analysis with H1 RNA as the loading control. In (**A**), RNA samples (20 µg) were isolated from cells with empty LXSN vector (P, lanes 3 and 6) and cells with EGS ICP8F (lanes 1 and 4) and ICP8I (lanes 2 and 5). Same samples were run on two identical gels, with one probed with the H1 RNA sequence and the other probed with the EGS RNA sequence. In (**B**), results are conveyed in % in comparison to those in cells with ICP8I and shown as mean ± SD. NS, not significant. Experiments were conducted in duplicate and repeated three times.

To generate EGS-expressing cell lines, DNA sequences encoding ICP8F and ICP8I were placed in retroviral vector LXSN and downstream from the U6 RNA promoter [23,24]. EGS-containing retroviruses were produced by transfection of amphotropic packaging cells (PA317) [22,25] with the constructed LXSN DNAs and then used to infect human foreskin fibroblasts (HFFs) to generate EGS-expressing cell lines. MTT assays revealed no substantial cytotoxicity among ICP8F- and ICP8I-expressing cells compared to cells with the empty LXSN vector for up to two months. EGSs were expressed at similar levels in the constructed cell lines examined in our Northern blot experiments with human H1 RNA as a loading control (Figure 2).

### 2.3. EGS Effects on Inhibiting HSV-2 ICP8 Gene Expression

**Figure 3:**
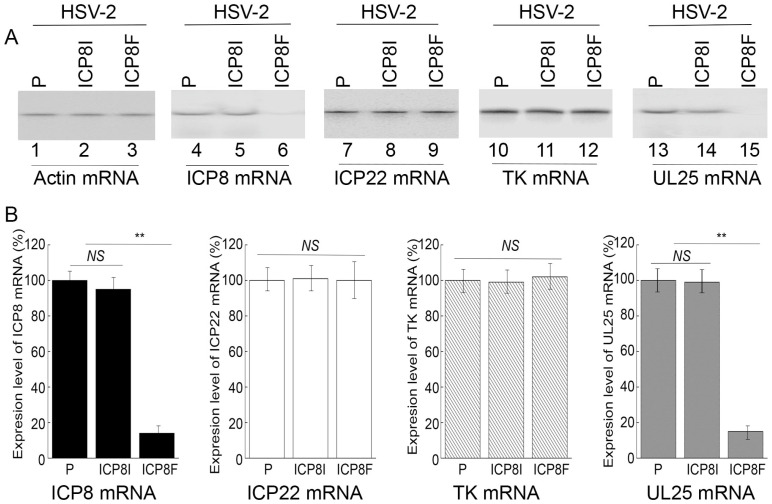
HSV-2 mRNA levels detected by Northern blot analyses with actin mRNA as the loading control. (**A**) Cells with the empty LXSN vector (P, lanes 1, 4, 7, 10, and 13) and cell lines with ICP8I (lanes 2, 5, 8, 11, and 14) and ICP8F (lanes 3, 6, 9, 12, and 15) were infected with HSV-2 (Multiplicity of Infection (MOI) = 1). RNAs were isolated at 4 h (lanes 7–9) or 10 h (lanes 1–6 and 10–15) post infection and hybridized to the RNA probes for actin mRNA (lanes 1–3), ICP8 mRNA (lanes 4–6), ICP22 (lanes 7–9), TK mRNA (lanes 10–12), and UL25 mRNA (lanes 13–15). (**B**) The mRNA levels of ICP8, ICP22, TK, and UL25 are conveyed in % in comparison to those in cells with the empty vector (P) and shown as mean ± SD. ** *p* < 0.05. NS, not significant. Experiments were conducted in duplicate and repeated three times.

**Figure 4:**
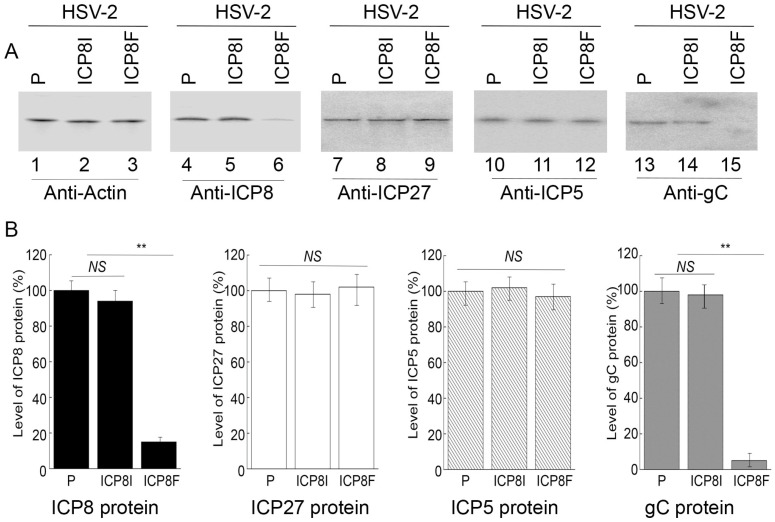
HSV-2 protein levels detected by Western blot analysis with actin protein as the loading control. (**A**) Cells with the empty LXSN vector (P, lanes 1, 4, 7, 10, and 13) and cell lines with ICP8I (lanes 2, 5, 8, 11, and 14) and ICP8F (lanes 3, 6, 9, 12, and 15) were infected with HSV-2 (MOI = 1). Protein samples were obtained at 6 h (lanes 7–9) or 12 h (lanes 1–6 and 10–15) post infection and reacted to antibodies against actin protein (lanes 1–3), ICP8 protein (lane 4–6), ICP27 protein (lanes 7–9), ICP5 protein (lanes 10–12), and gC protein (lanes 13–15). (**B**) The protein levels of ICP8, ICP27, ICP5, and gC are conveyed in % in comparison to those in cells with the empty vector (P) and shown as mean ± SD. ** *p* < 0.05. NS, not significant. Experiments were conducted in duplicate and repeated three times.

Levels of ICP8 mRNA and protein were quantified by Northern and Western blot analyses with the actin mRNA and protein as the loading controls, respectively (Figure 3 and Figure 4). In our experiments, RNA and protein samples were isolated from HSV-2 infected cells with the empty LXSN vector or EGS ICP8F and ICP8I at different times post infection. A decrease of ~86% and 85% in ICP8 mRNA and protein levels, respectively, was detected in the cells with ICP8F (Figure 3 and Figure 4). On the contrary, no significant reduction in ICP8 mRNA and protein expression was observed in the cells with the control EGS ICP8I. These results implied that the RNase P cleavage of ICP8 mRNA guided by ICP8F might be the cause contributing to the substantial inhibition of ICP8 expression in the cells with ICP8F. This is because ICP8I shared the same antisense sequences against sub-icp8 as ICP8F but was not active in guiding RNase P cleavage due to the three nucleotide mutations at the T-loop region (Figure 1 and Table 1). No intracellular cleavage products of ICP8 mRNA were found, possibly because these RNAs were unstable and quickly hydrolyzed intracellularly due to their lack of a 5′ cap structure or a poly(A) sequence.

### 2.4. Antiviral Effects of the EGSs in Inhibiting HSV-2 Growth

**Figure 5:**
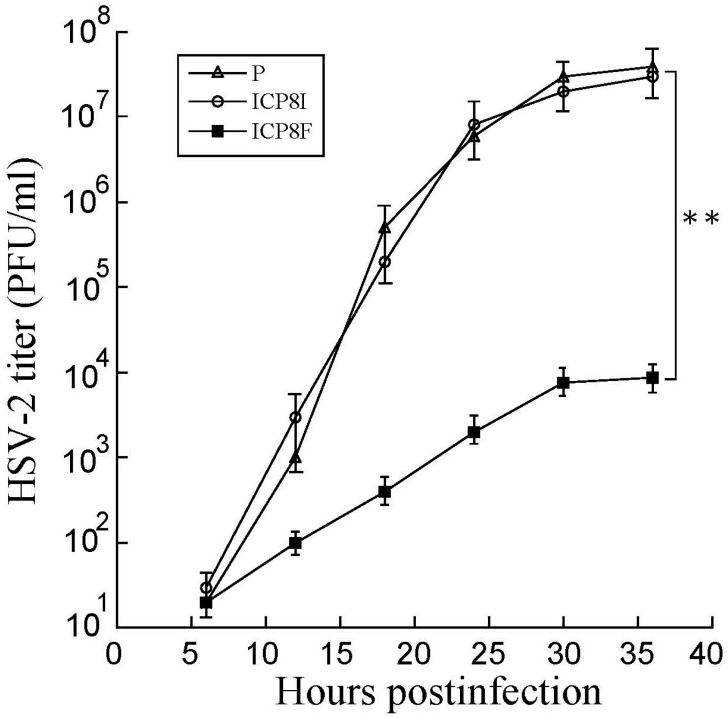
Growth analysis of HSV-2 in cells with the empty LXSN vector (P), ICP8I, and ICP8F. HSV-2 stocks were prepared from the cells (infected with HSV-2 at an MOI of 0.8), and culture media harvested every 6 h for 36 h post infection. We determined the virus titer by assaying the plaque forming unit (PFU) in Vero cells. The results are shown as mean ± SD. ** *p* < 0.05. Experiments were conducted in duplicate and repeated three times.

Decreased ICP8 expression could result in reduced HSV-2 growth because this protein is essential for viral DNA genome replication and lytic infection [1,17]. EGS ICP8F seemed to suppress viral growth in HSV-2 infected cells. Cells with the empty LXSN vector and EGS ICP8F and ICP8I were infected with HSV-2 (Multiplicity of Infection (MOI) = 1). Virus stocks were obtained from cells and culture media collected at various times post-infection and titered. After 18 h post infection, virus yield was inhibited by more than 3000 folds in cells with ICP8F (Figure 5). In contrast, we found no substantial inhibition in cells with the control EGS ICP8I. Thus, ICP8F effectively blocked viral growth. These results also imply that RNase P cleavage of ICP8 mRNA guided by the functional ICP8F is responsible for the observed antiviral effect, as the control EGS ICP8I, which shared an identical binding sequence to the ICP8 mRNA as ICP8F but had no targeting activity (Figure 1 and Table 1), exhibited little antiviral effect.

### 2.5. EGS Effects in HSV-2 Gene Expression and Genome Replication

**Figure 6:**
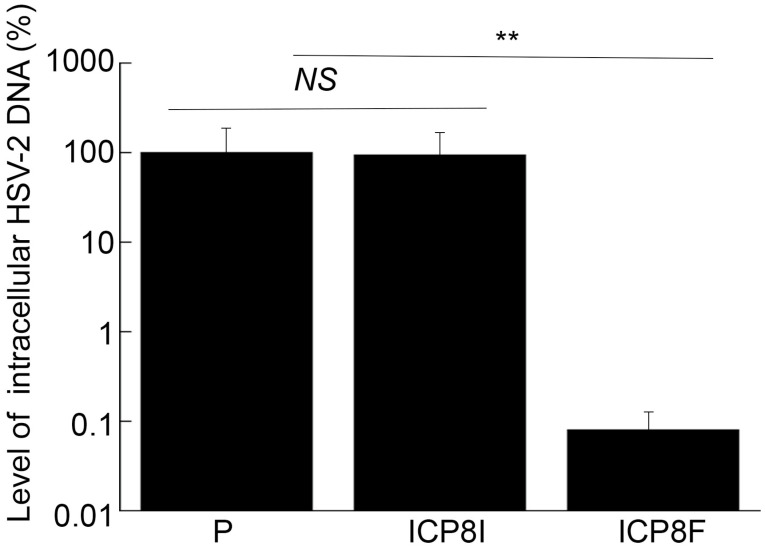
Intracellular HSV-2 DNA levels quantified by qPCR with actin DNA as the loading control. Cells with the empty LXSN vector (P), ICP8I, and ICP8F were infected with HSV-2 (MOI = 1). Total DNAs were isolated at 11 h post infection and quantified using qPCR, which amplified the ICP4 DNA sequence. The results are conveyed in % in comparison to those in cells with the empty vector (P) and shown as mean ± SD. ** *p* < 0.05. NS, not significant. Experiments were conducted in duplicate and repeated three times.

ICP8 is expressed during the early phase (E) of HSV-2 infection and encodes a single-stranded DNA binding protein that is essential for viral DNA genome replication [1,17]. Inhibition of ICP8 expression is expected to block HSV-2 DNA genome replication, which should not affect the expression of viral immediate-early (IE) and early (E) genes but decreases the expression of viral late (L) genes [1,17]. To determine if this is the case, two series of experiments were conducted. In the first series of experiments, total DNA was extracted from cells infected with HSV-2, and the levels of the HSV-2 genome were quantified using qPCR, which amplified the HSV-2 ICP4 DNA sequence. The level of human actin DNA was chosen as the loading internal control. It is known that the HSV-2 genomic DNA remains episomal and does not integrate into the host genome [1,17]. Thus, the amount of intracellular viral DNA truly reflects the viral genome level. We found no remarkable difference in the levels of total intracellular HSV-2 DNA in the cells with the empty LXSN vector and with the control EGS ICP8I (Figure 6). Rather, we found that the levels of intracellular HSV-2 DNA decreased by 1000 folds in the cells with functional EGS ICP8F, indicating the blockage of HSV-2 genome replication in these cells as a result of the ICP8F-mediated reduced ICP8 expression. Moreover, the suppression of HSV-2 genome replication by ICP8F is due to the ICP8F-guided RNase P cleavage of ICP8 mRNA because no changes in viral DNA genome replication were found in the cells with the control EGS ICP8I, which contained the same targeting sequence to ICP8 mRNA as ICP8F but exhibited little targeting activity.

In the second series of experiments, we studied the effects of the EGS expression on the mRNA and protein expression of different HSV-2 genes, including the viral immediate-early (IE), early (E), and late (L) genes. Specifically, Northern and Western blot analyses were employed to assay the expression of ICP22 mRNA (an IE gene), ICP27 protein (an IE gene), TK mRNA (UL23, an E gene), ICP5 protein (an E gene), UL25 mRNA (a late gene), and gC protein (a late gene) with the actin mRNA and protein as the loading controls, respectively (Figure 3 and Figure 4). We found no substantial difference in the expression of viral IE and E genes examined, including ICP22 mRNA, ICP27 protein, TK mRNA, and ICP5 protein, in the cells with the empty LXSN vector or with different EGSs. On the contrary, a decrease of 85% in the mRNA level of UL25, a late gene, was detected in the cells with ICP8F (Figure 3). A reduction of about 95% was also detected in the level of viral glycoprotein gC, another late gene product (Figure 4). We noted that no significant decrease in the expression of these late genes was shown in the cells with ICP8I compared to the cells with the empty LXSN vector (Figure 3 and Figure 4).

The salient features and implications of these results are three-fold. First, these results revealed that ICP8F suppressed the expression of ICP8 (an early gene) but had no effect on the expression of the viral immediate-early and early genes examined, suggesting that ICP8F is specific in targeting ICP8 mRNA but not the mRNAs of other viral IE or E genes. Second, EGS ICP8F decreased the expression of the viral late genes examined (i.e., UL25 mRNA and gC protein), probably resulting from the inhibition of HSV-2 DNA genome replication due to the ICP8F-mediated suppression of ICP8 expression. Finally, our results imply that the RNase P-mediated cleavage of ICP8 mRNA guided by ICP8F accounts for the observed suppression of ICP8 expression, reduction in viral DNA genome replication, and decrease in viral late gene expression. This implication stems from our results that no changes in viral gene expression and genome DNA replication were found in the cells with the control EGS ICP8I, which had identical targeting sequences to ICP8 mRNA as functional EGS ICP8F but showed little targeting activity.

## 3. Discussion

Nucleic acid-based gene interference and editing approaches, including ribozymes, small interfering RNAs (siRNAs), and guide RNAs for CRISPR/cas9, represent promising methods for gene targeting applications, including therapy of various human diseases [3,4,5,6]. Among these approaches, the RNase P-EGS technology represents a unique and attractive gene targeting approach due to its use of endogenous RNase P to generate highly efficient and specific cleavage of the target RNA. Because RNase P is accountable for processing all tRNA molecules in a human cell, this enzyme is believed to be very active in vivo [8]. Moreover, previous studies have shown that RNase P-mediated cleavage guided by EGSs is specific and does not generate the irrelevant cleavage commonly associated with RNase H-mediated cleavage induced by conventional antisense phosphothioate oligonucleotides [26]. Our study here represents the first investigation to use the RNase P-EGS technology for anti-HSV-2 applications.

In this report, a functional EGS, ICP8F, was developed to target the HSV-2 ICP8 mRNA. In vitro, ICP8F efficiently guided human RNase P in cleaving the ICP8 mRNA substrate sub-icp8. Cultured cells expressing ICP8F demonstrated an 85–86% reduction in ICP8 expression and a 3000-fold decrease in HSV-2 growth. Meanwhile, cells expressing the control inactive EGS ICP8I showed a 5–8% reduction in ICP8 expression and no reduction in HSV-2 growth. Inactive EGS ICP8I demonstrated little targeting activity due to the T-loop mutations precluding RNase P recognition. This EGS contained an identical guide sequence and had a similar binding affinity to the substrate sub-icp8 as functional EGS ICP8F. Thus, our results suggest that the suppression of viral gene expression and growth with functional EGS ICP8 was due to the RNase P-mediated cleavage of the ICP8 mRNA induced by the EGS, as opposed to the antisense effect or other non-specific effects of the guide sequence of the EGSs.

Several lines of evidence indicated that ICP8F-guided cleavage of the ICP8 mRNA by human RNase P appeared to be specific. First, cells with EGSs demonstrated no difference from those without EGSs in terms of cell growth and viability for up to two months, implying that the expression of the EGSs was not toxic to cells. Second, EGS ICP8F specifically suppressed ICP8 (an early gene) expression but not the expression of other HSV-2 immediate-early and early genes such as ICP22 (an IE gene), ICP27 (an IE gene), TK (an E gene), and ICP5 (and E gene). We detected no reduction in the mRNA levels of ICP22 and TK (Figure 3) or the protein levels of ICP27 and ICP5 (Figure 4) in the ICP8F-expressing cells. Thus, ICP8F specifically targeted against its target, the ICP8 mRNA, but not other viral mRNAs. Third, the antiviral effects (inhibition of viral growth and viral gene expression) in the cells with ICP8F and associated with ICP8F expression seemed to result from a suppression of ICP8 expression. We observed a decrease in viral late gene expression (e.g., UL25 and gC) in the cells with ICP8F but not in those with the control EGS ICP8I (Figure 3 and Figure 4). The extent of the decrease in HSV-2 late gene expression and growth correlates with the decrease in inhibition of the ICP8 expression. These results are consistent with the notion that ICP8 protein is required for viral genome replication, which is important for viral late gene expression [1,17]. Thus, ICP8F-guided cleavage of the ICP8 mRNA by human RNase P appears to be specific and responsible for the antiviral effect of the EGS.

HSV-2 belongs to the human herpesvirus family, which contains additional human viruses, including herpes simplex virus 1 (HSV-1), cytomegalovirus, Epstein-Barr virus, varicella-zoster virus, and Kaposi’s sarcoma-associated herpesvirus [27]. HSV-1 shares an overall genome sequence homology of about 50% with HSV-2 and causes unique human diseases such as cold sores [1,2]. We previously constructed EGSs [16] to target the mRNA of HSV-1 ICP4, which encodes a transcription activator required for viral gene transcription [1,17]. These EGSs blocked HSV-1 ICP4 expression, leading to overall inhibition of viral gene expression and viral growth in cultured cells [16]. In the current study, EGSs were constructed to target HSV-2 ICP8. The ICP8 protein is highly conserved among all human herpes viruses and encodes the single-stranded DNA binding protein required for viral DNA genome replication [1,17]. Our effective suppression of HSV-2 ICP8 expression and growth presented in this study implies that targeting ICP8 and its homologs in other herpesviruses by human RNase P and EGSs may represent a promising approach for antiviral therapy against these herpesviruses.

All herpesviruses, including HSV-2, have two modes of infection: lytic replication and latent infections [27]. To further develop the EGS approach against HSV-2, one challenge is the delivery of EGSs to neuronal cells, in which viral latent infection is established [1,17]. It will be important to investigate the antiviral effects of the delivered EGSs and determine if the EGSs can block virus reactivation in these cells. With these additional investigations, we aim to develop the EGS technology as a novel and promising tool for anti-HSV-2 therapy.

## 4. Materials and Methods

### 4.1. Antibodies, Viruses, and Cells

Human foreskin fibroblasts (HFFs), Vero, and PA317 cells (purchased from American Type Culture Collection, Manassas, VA, USA) were cultured following protocols as described previously [23,28]. Infection and production of HSV-2 (G strain) were conducted as described previously [29,30]. Antibodies against human actin and various HSV-2 proteins were obtained from Virusyn (Taneytown, MD, USA), Promab Inc (Richmond, CA, USA), Abcam (Waltham, MA, USA), and Sigma Inc (St Louis, MO, USA).

### 4.2. EGS Studies In Vitro

We constructed the ICP8F coding sequence by PCR with the 5′ primer 5-ICP8F (5′-GGAATTCTAATACGACTCACTATAGGTTAACATACACGGTGCGGTCTCCGCGC-3′) and the 3′ primer 3-ICP8F (5′-AAGCTTTAAATGATGGGGGCAGGATTTGAACCTGCGCGCGGAGACCGCAC-3′). Primers 5-ICP8F and 3-ICP8F were allowed to anneal to each other, and the annealed products were amplified by PCR to generate the DNA sequence encoding EGS ICP8F. We constructed the control ICP8I coding sequence by PCR using the ICP8F coding sequence as the template with the 5′ primer 5-ICP8I (5′-GGAATTCTAATACGACTCACTATAG-3′) and the 3′ primer 3-ICP8I (5′-AAGCTTTAAATGATGGGGGCAGGATTTCTTCCTGCGCGCGGAGACCGCAC-3′). The 3′ primer 3-ICP8I contained the three nucleotide mutations in the T-loop region of the EGS (Figure 1C).

We generated the sub-icp8 coding sequence by annealing the oligonucleotide AF25 (5′-GGAATTCTAATACGACTCACTATAG-3′) and the oligonucleotide Sub-icp8-3 (5′-CGGATCCCGGCCATACACGTACCCCAUCGGCCCCGGCCCCTATAGTGAGTCGTATTA-3′). The oligonucleotide AF25 contained the T7 RNA polymerase promoter, and the oligonucleotide Sub-icp8-3 contained the coding sequence for the substrate sub-icp8. The annealed products of these two oligonucleotides were used as the templates for in vitro transcription to generate the RNA substrate sub-icp8.

The EGS and sub-icp8 RNAs were in vitro produced with T7 RNA polymerase (Promega, Inc., Madison, WI, USA). We assayed kinetic parameters in reactions with human RNase P (extracted from HeLa cells) in buffer A (10 mM MgCl_2_, 50 mM Tris, pH 7.4, 100 mM NH_4_Cl) as described previously [13,14,21,22]. Binding affinities were also assessed following previously described procedures [20,31].

### 4.3. EGS Expression in Cells

We followed published protocols for constructing EGS-expression [23,24]. PA317 cells were transfected with retroviral LXSN vectors with no EGS or different EGS sequences (i.e., LXSN, LXSN-ICP8F, and LXSN-ICP8I). Human foreskin fibroblasts (HFFs) were infected with the supernatants from the PA317 cells, selected, and cloned with neomycin (500 µg/mL). Northern blot analysis was conducted to quantify the EGS levels as described previously [23,24,32,33]. In brief, RNAs were isolated, separated in formaldehyde-containing gels, transferred to membranes, hybridized with radiolabeled probes containing H1 RNA and EGS sequences, and analyzed using a STORM 840 Phosphorimager (Molecular Dynamics, Sunnyvale, CA, USA) [32,33].

### 4.4. Assessing HSV-2 Infection, Gene Expression, Genome Replication, and Growth

Cells were either mock-infected or infected with HSV-2 (MOI = 0.5–1). In the experiments with viral mRNAs, we collected total RNA at 4 or 10 h post infection. In the experiments with viral proteins, we collected cell proteins at 6 or 12 h post infection. The levels of the mRNAs and proteins were quantified with Northern and Western blot analyses, respectively, as described previously [32,33]. In the Northern blot analyses, RNAs were isolated, separated in formaldehyde-containing gels, transferred to membranes, hybridized with radiolabeled probes containing the sequences for human actin and different HSV-2 mRNAs, and analyzed with a STORM 840 Phosphorimager. In the Western blot analysis, proteins were isolated, separated in denaturing gels, transferred to membranes, reacted with primary antibodies against actin and different HSV-2 proteins, immunoreacted with HRP-conjugated secondary antibody, stained with the ECL substrates (GE Healthcare, Chicago, IL, USA), and then analyzed with a STORM 840 Phosphorimager [32,33].

In the experiments evaluating the antiviral effects of the EGS, we obtained virus stocks from the cells and collected culture media every 6 h for 36 h post infection. We quantified the samples for their virus titers by performing plaque assays in Vero cells. The experiments were conducted in duplicates and repeated three times.

In the experiments evaluating the effects of the EGS on HSV-2 genome replication, we purified total DNAs at 11 h post infection and quantified the DNA levels with a qPCR assay including SYBR Green and amplifying the viral ICP4 sequence. We performed the PCR reactions (Bio-Rad iCycler) with ICP4 sequence-specific primers (5′ primer: GCGTCGTCGAGGTCGT, 3′ primer: CGCGGAGACGGAGGAG) in a Bio-Rad (Hercules, CA, USA) PCR mix [21,34,35]. We quantified the actin DNA levels, which served as the control, by performing PCR with the 5′ primer Actin5 (5′-TGACGGGGTCACCCACACTGTGCCCATCTA-3′) and the 3′ primer Actin3 (5′-CTAGAAGCATTGCGGTGGCAGATGGAGGG-3′) [21,34,35].

### 4.5. Statistical Analysis

We conducted the assays in duplicate and repeated them three times. The data were analyzed with GraphPad Prism software (version 9) and statistical analyses were performed with the analysis of variance (ANOVA). We considered differences with *p* < 0.05 as statistically significant.

## 5. Conclusions

We showed that an EGS induced RNase P to degrade HSV-2 ICP8 mRNA in vitro and suppressed ICP8 expression and viral growth in cultured cells. Our study provides the first direct evidence that EGSs are effective and specific anti-HSV-2 agents. Moreover, this study demonstrates the potential use of RNase P complexed with an EGS as a promising gene targeting approach for anti-HSV-2 therapy.

## Figures and Tables

**Table 1:** Overall cleavage rates (V_max(apparent)_/K_m(apparent)_) and binding affinities (K_d_) in EGS-mediated cleavage reactions of the ICP8 mRNA substrate sub-icp8 by RNase P (from HeLa cells). Experiments were conducted in duplicate and repeated three times, as described previously [13,14,21,22].

Substrate (sub-icp8)	K_m_ (µM)	V_max (apparent)_ (pmol·min^−1^)	V_max(apparent)_/K_m(apparent)_ (pmol·µM^−1^·min^−1^)	K_d_ (µM)
+EGS ICP8F	0.31 ± 0.11	0.025 ± 0.006	0.081 ± 0.012	1.5 ± 0.3
+EGS ICP8I	ND	ND	ND	1.5 ± 0.3

“ND”: not determined.

## Data Availability

Data are contained within the article.
